# CULTURAL AND PSYCHOMETRIC VALIDATION FOR COGNITIVE ASSESSMENT OF OLDER ADULTS

**DOI:** 10.1093/geroni/igae098.3944

**Published:** 2024-12-31

**Authors:** Rita Bárbara, Rui Gonçalves, Mario Lopes

**Affiliations:** Universidade de Aveiro, AVEIRO, Aveiro, Portugal; Escola Superior de Tecnologia da Saúde de Coimbra do Instituto Politécnico de Coimbra., Coimbra, Coimbra, Portugal; Universidade de Aveiro, AVEIRO, Aveiro, Portugal

## Abstract

**Introduction:**

Cognitive assessment is vital for the early detection and management of cognitive disorders in older adults. Instruments developed in one cultural context may not be applicable to other populations without proper adaptation. This study aims to culturally adapt the cognitive domain of the Older Americans Resources and Services (OARS) instrument for use with Angolan elderly, ensuring it accurately captures the cultural and specific needs of this group. Objective: The primary goal is to validate the cognitive domain of the OARS for its cultural relevance and clinical reliability in Angola. This involves modifying the instrument to better suit the Angolan cultural context.

**Methods:**

The study included 127 Angolan older adults. An initial factor analysis identified items misaligned with the local cultural context. These items were revised and adapted. A follow-up factor analysis evaluated the coherence of the revised instrument. Reliability was measured using Cronbach’s alpha.

**Results:**

The initial analysis revealed several problematic items, which were modified for improved cultural fit. The revised factor analysis demonstrated a more coherent structure, with Cronbach’s alpha rising to 0.82, indicating good internal consistency among the updated items.

**Discussion:**

Culturally adapting the OARS was crucial for improving its sensitivity to Angolan older adults’ needs, leading to a more accurate and equitable cognitive assessment.

**Conclusion:**

The adaptation and psychometric validation of the OARS have produced a more suitable tool for cognitive assessment in Angola, highlighting the importance of considering cultural factors in instrument development. Keywords: Cognitive assessment, cultural adaptation, elderly.

